# Detection of up to 65% of Precancerous Lesions of the Human Colon and Rectum by Mutation Analysis of *APC, K-Ras, B-Raf* and *CTNNB1*

**DOI:** 10.3390/cancers3010091

**Published:** 2010-12-29

**Authors:** Mandy Schneider, Bettina Scholtka, Uwe Gottschalk, Siegbert Faiss, Daniela Schatz, Kornelia Berghof-Jäger, Pablo Steinberg

**Affiliations:** 1 Chair of Nutritional Toxicology, Institute of Nutritional Science, University of Potsdam, Arthur- Scheunert-Allee 114-116, 14558 Nuthetal, Germany; E-Mail: manschne@uni potsdam.de; 2 Maria Heimsuchung Caritas-Klinik Pankow, Breite Straße 46/47, 13187 Berlin, Germany; E-Mail: uwe.gottschalk@caritas-klinik-pankow.de; 3 III. Medizinische Abteilung - Gastroenterologie und Hepatologie, Asklepios Klinik Barmbek, Rubenkamp 220, 22291 Hamburg, Germany; E-Mail: s.faiss@asklepios.com; 4 BIOTECON Diagnostics GmbH, Hermannswerder Haus 17, 14473 Potsdam, Germany; E-Mails: daniela.schatz@online.de (D.S.); kberghof@bc-diagnostics.com (K.B.-J.); 5 Institute for Food Toxicology and Analytical Chemistry, University of Veterinary Medicine Hannover, Bischofsholer Damm 15, 30173 Hannover, Germany

**Keywords:** adenomas, *APC*, *B-Raf*, *CTNNB1*, gene mutations, human colon, serrated lesions, *K-Ras*, primer panel

## Abstract

In the present study a recently conceived 4-gene marker panel covering the Wnt and Ras-Raf-MEK-MAPK signaling pathways was used to analyze 20 colorectal serrated lesions and 41 colorectal adenoma samples and to determine the percentage of each of the above-mentioned potentially precancerous lesions carrying at least one of the four above-mentioned genes in a mutated form. *CTNNB1* and *B-Raf* were screened by PCR-single-strand conformation polymorphism analysis, *K-Ras* by restriction fragment length polymorphism analysis and the *APC* gene mutation cluster region (codons 1243–1567) by direct DNA sequencing. *APC* mutations were only detected in 10% of the serrated lesions but in 34% of the adenomas. Twenty percent of the serrated lesions and 14% of the adenomas carried a mutated *K-Ras*. *B-Raf* was found to be mutated in 50% of the serrated lesions and in 22% of the adenomas. *CTNNB1* was altered in 12% of the adenomas, but not in serrated lesions. By using the above gene marker panel it could be shown that 65% of the serrated lesions and 61% of the adenomas carried at least one of the four genes in a mutated form. Based on its excellent performance in detecting mutations in sporadic preneoplastic (in this study) and neoplastic lesions (in a previous study) of the human colon and rectum, this primer combination might also be suited to efficiently and non-invasively detect genetic alterations in stool DNA of patients with early colorectal cancer.

## 1. Introduction

Sporadic colorectal cancer (CRC) is one of the leading cancer diseases in the Western world. CRC is the result of a multistep process. In the course of this process a number of genetic alterations accumulate, which then lead to the malignant transformation of epithelial cells in the colon or rectum. The “traditional” pathway, the so-called adenoma-carcinoma-sequence, described by Vogelstein and colleagues in the early 1990s, is characterized by an early bi-allelic inactivation of *APC* caused by alterations in the mutation cluster region (codons 1243–1567) of this gene followed by an oncogenic *K-Ras* codon 12 or 13 mutation, inactivation of the tumor suppressor gene *Tp53* at the transition from adenoma to carcinoma [1-3] and chromosomal instability [4]. In accordance with this model, adenomas and even single aberrant crypt foci, the earliest putative CRC precursors, were shown to harbour mutations in the *APC* gene [3,5]. Furthermore, Sparks *et al.* [6] suggested that mutations in *CTNNB1*, which, like *APC*, is involved in the Wnt signaling pathway and encodes the protein β-catenin, could uniquely substitute *APC* mutations at early stages of CRC development. Later studies reported that *CTNNB1* mutations can be observed in CRC [7-9], although at a significantly lower frequency than that originally reported by Sparks *et al.* [6].

In recent years, an alternative concept to the above-mentioned adenoma-carcinoma sequence, the so-called serrated pathway, in which a subgroup of serrated lesions (so called microvesicular hyperplastic polyps and sessile serrated adenomas) give rise to serrated carcinomas, has been postulated [10-13]. At the molecular level, serrated carcinomas are characterized by missense mutations of *B-Raf* codon 600, and extensive DNA methylation. Microsatellite instability (MSI) is common as well [12].

Jass *et al.* [14] explored the possibility that the early evolution of CRC is not limited to the adenoma-carcinoma-sequence and the serrated adenoma pathway, but often combines components of both pathways in a so-called fusion pathway. Specifically, it was suggested that methylation of the DNA repair gene *O-6-methylguanine DNA methyltransferase*, mutation of *K-Ras* and inactivation of the tumor suppressor gene *Tp53*, provide critical combinations of molecular “cross-over” between the two pathways at the stage of precancerous polyps. Thus, *B-Raf* and *K-Ras* mutations, which are supposed to be mutually exclusive and are both involved in the Ras-Raf-MEK-MAPK signaling pathway (MAPK, mitogen-activated protein kinase; MEK, MAPK/ERK kinase), represent early steps in tumor development [15-17].

Although CRC develops over a very long period of time, about two thirds of the patients already have advanced cancer with poor prognosis upon diagnosis. Even for patients with UICC stage II carcinomas the long-term overall survival is still quite low [18]. Therefore, highly sensitive screening methods, which are not only able to detect early (stage I) carcinomas but also precancerous lesions and which are hopefully better accepted by the patients than colonoscopy, are urgently needed. Non-invasive screening for molecular CRC markers could be an option in this context. Hence, a thorough selection of appropriate markers is required for this purpose.

A new set of oligonucleotide primers that allows the analysis of the mutational hotspots within the genes coding for APC, β-catenin, B-Raf and K-Ras was recently conceived [19]. These four genes were selected because they are involved in the Wnt and the Ras-Raf-MEK-MAPK signaling cascades and therefore play a substantial role in the adenoma-carcinoma as well as in the serrated adenoma pathway. By making use of this primer panel it could very recently be shown that 74–80% of the analyzed early stage CRC (UICC stages I and II) and over 80% of CRC in UICC stage IV carried at least one of the four above-mentioned genes in a mutated form [20].

In the present study, the primer combination mentioned above was used to analyze the frequency of *APC*, *K-Ras*, *B-Raf* and *CTNNB1* mutations in precancerous lesions (serrated lesions and adenomas) from human colon and rectum. These analyses allowed to determine whether alterations in the Wnt pathway and in the Ras-Raf-MEK-MAPK pathway occurred simultaneously in each individual lesion and to define the relative importance of both pathways in the development of CRC.

## 2. Results

In this prospective pilot study on precancerous human colonic lesions, 20 serrated lesions, 41 colorectal adenomas and 10 controls (biopsies of inflamed colon mucosa), were analyzed for gene mutations. Clinicopathologic data of the colorectal samples analyzed in the present study are shown in Table 1. Thirty samples were located in the proximal colon (including caecum, ascending colon, hepatic flexure, transverse colon and splenic flexure), 23 in the distal colon (descending colon and sigmoid colon) and 11 in the rectal part of the gastrointestinal tract (rectosigmoid junction and rectum). Thirty-five adenomas were typified as tubular and four as tubulovillous. Thirty-six adenomas exhibited a low grade and three a high grade dysplasia.

In a previous study on CRC (UICC stages I–IV) [20] *B-Raf* and *CTNNB1* mutations were successfully detected by single strand conformation polymorphism (SSCP) analysis, *K-Ras* mutations by restriction fragment length polymorphism (RFLP) analysis and *APC* mutations by direct sequencing of the mutation cluster region. In order to be able to compare the data for the preneoplastic lesions with the results for CRC [20], the same three methods were used. The results of the gene mutation analysis of all samples included in this study are presented in the Electronic Supplementary Information (Table S1). All mutations in *K-Ras, B-Raf* and *CTNNB1* were single base substitutions, which led to the exchange of an amino acid in the corresponding gene product. In the case of *K-Ras*, codon 12 was found to be mutated in three serrated lesions and in two adenomas. Mutations in codon 13 of *K-Ras* were detected in one serrated lesion and in five adenomas. A single adenoma carried a base substitution in codon 12 as well as in codon 13 of *K-Ras*. Mutations in the *B-Raf* gene were restricted to codon 600 and were present in ten serrated lesions and in nine adenomas. Regarding *CTNNB1*, three adenomas carried the codon 41 ACC→GCC mutation and two adenomas exhibited the codon 45 TCT→TTT mutation. The five single base substitutions detected in the *APC* gene resulted in four cases in a stop codon leading to protein truncation and in one case in an amino acid exchange. Deletions in *APC* were detected in six cases (one serrated lesion and five adenomas); insertions in five adenomas. Codon 1465 of the *APC* gene (deletion of an AG) was found to be modified in one serrated lesion and in two adenomas, while an insertion of a single A in codon 1556 was detected in two adenomas. All determined deletions or insertions caused frameshifts. In a single adenoma, three genes (*APC*, *K-Ras* and *CTNNB1*) were found to be altered. Four samples (one serrated lesion and three adenomas) showed alterations both in *APC* and in *B-Raf* and three samples (two serrated lesions and one adenoma) in *K-Ras* as well as in *B-Raf*. In a single adenoma, *APC* as well as *CTNNB*, and in two adenomas *APC* as well as *K-Ras*, were altered.

The gene mutation frequencies for each kind of the examined lesions are summarized in Table 2. No mutations were detected in the inflamed tissue samples. Whereas alterations in the *APC* mutation cluster region were only observed in ten percent of the serrated lesions, 34.1% of the adenomas exhibited mutations. Moreover, twenty percent of the serrated lesions and 14.6% of the adenomas carried a mutated *K-Ras* gene. Of the four genes analyzed *B-Raf* was the most frequently mutated, 50% of the serrated lesions and 22% of the adenomas being altered. *CTNNB1* was mutated in 12.2% of the adenomas, but not in serrated lesions. By making use of our oligonucleotide primer panel for the PCR-based mutation analysis of the four marker genes, it could be shown that at least one of the four genes analyzed was altered in 65% of the serrated lesions and in 61% of the adenomas.

The proteins APC and β-catenin are involved in the Wnt signaling pathway, whereas B-Raf and K-Ras are proteins associated with the Ras-Raf-MEK-MAPK signaling cascade. In serrated lesions the Ras-Raf-MEK-MAPK pathway was significantly more often altered than the Wnt pathway (60% *versus* 10%, Table 3). The Wnt and Ras-Raf-MEK-MAPK pathways were affected in 41.5 and 34.2% of the adenomas, respectively. Taken together, 13 out of 20 serrated lesions (65%) and 25 out of 41 adenomas (61%) exhibited alterations in one or both signaling pathways.

Interestingly, 12 out of 13 altered serrated lesions (92.3%) and 19 out of 25 altered adenomas (76%) carried mutations in only one of the two above-mentioned signaling pathways (Table 3). In 11 of 12 serrated lesions (91.7%), in which only one pathway was altered, a gene mutation in the Ras-Raf-MEK-MAPK pathway was detected, while in the remaining sample the Wnt pathway (*APC*) was affected. In 11 of 19 adenomas (57.9%), in which only one pathway was altered, genes being part of the Wnt pathway were mutated, whereas in the remaining eight adenoma cases (42.1%) the Ras-Raf-MEK-MAPK pathway was affected.

In a further step, the correlation between the anatomical location of serrated lesions and adenomas, and the frequency of genetic alterations in both signaling pathways, was evaluated. In serrated lesions mutations of the Wnt signaling pathway were extremely rare and were limited to lesions arising in the distal colon (Table 4). In contrast, mutations in the Ras-Raf-MEK-MAPK pathway were more frequently detected in serrated lesions, these being predominantly observed in the proximal colon. The frequency of *B-Raf* and *K-Ras* mutations in serrated lesions decreased from the proximal colon towards the rectum. In the case of adenomas the proportion of Wnt pathway alterations tended to be higher in those lesions arising in the distal colon and rectum, whereas mutations in *K-Ras* and *B-Raf* were predominantly present in adenomas of the proximal colon. In adenomas arising in the distal colon, genes involved in the Wnt pathway were more frequently mutated than those in the Ras-Raf-MEK-MAPK pathway.

## 3. Discussion

By making use of a recently described primer panel [19] it could be shown that in up to 65% of the potentially precancerous lesions analyzed, at least one of the four genes studied was altered. *B-Raf* was frequently mutated in serrated lesions (50%), as well as in traditional adenomas (22%). These percentages are within the range of those obtained in previous studies [11,14-16,21-24] and support the hypothesis that *B-Raf* mutations occur at an early stage of CRC development. In the case of *APC* only 10% of the serrated lesions analyzed carried a mutated gene. If one takes into account the study by Fogt *et al.* [22], in which the hyperplastic polyps analyzed showed no loss of heterozygosity in the case of *APC*, and the report by Zauber *et al.* [25], in which no *APC* mutations were detected in serrated lesions, one must conclude that at the very early stage of colorectal carcinogenesis *APC* mutations are rare. However, they become frequent in colorectal adenomas as demonstrated by the fact that in the present study a mutated *APC* gene was detected in one third of the adenoma samples analyzed. *K-Ras* was found to be mutated in 20 and 14.6% of the serrated lesions and traditional adenomas, respectively. Hence, the *K-Ras* mutation frequencies observed are within the same range as those obtained in previous reports [14-16]. *CTNNB1* mutations were not detected in serrated lesions. Furthermore, the mutation frequency of *CTNNB1* was low in adenomas (12.2%), a finding that is in accordance with most of the previous analyses on colorectal adenomas [26-28] and with the observation that *CTNNB1* mutations mainly occur in hereditary nonpolyposis colorectal cancer [29] and in tumors exhibiting MSI [7,30]. The results obtained in the present report also show that in about one third of the serrated lesions and adenomas analyzed neither the Wnt nor the Ras-Raf-MEK-MAPK signaling pathway are affected, thus suggesting that the above-mentioned lesions might develop along a pathway different from the so-called adenoma-carcinoma sequence [1-3], the serrated adenoma pathway [10-12,17], or the fusion pathway [14]. In the present study inflamed tissue samples but no initial stage of benign proliferative tissue (e.g. hyperplastic mucosa or aberrant crypt foci) were included in the analysis. Thus, at the present time one cannot determine whether such lesions may also carry single gene mutations.

Interestingly, in 92.3% of the mutation-positive serrated lesions and in 76% of the adenoma samples with mutations, only one of the two signaling cascades analyzed was altered. The Ras-Raf-MEK-MAPK signaling cascade was solely affected in 92.3 and 56% of the serrated lesion and adenoma cases with mutations, respectively, whereas the Wnt signaling cascade was solely mutated in 15.4 and 68% of the serrated lesion and adenoma cases with mutations, respectively. Moreover, in a recent study by our group, in which CRC samples underwent a mutational analysis with the same oligonucleotide primer panel used in the present report, it was shown that 45% of mutation-positive CRC only exhibited alterations in the Wnt signaling cascade and 38% of mutation-positive CRC only exhibited alterations in the Ras-Raf-MEK-MAPK signaling cascade [20]. Thus, alterations in the Wnt pathway alone seem to confer a certain number of lesions with a growth/selection advantage, whereby alterations of the Ras-Raf-MEK-MAPK signaling cascade in the absence of alterations in the Wnt pathway can also act as a driving force in the multistep process of colon carcinogenesis. The above-mentioned assumption is strongly supported by the data of a very recent oncogenetic tree analysis performed on 971 colon tumors by Sweeney *et al.* [31]. The oncogenetic tree is a model intended to describe the pathways and sequence of somatic alterations in carcinogenesis without assuming that tumors will fall in mutually exclusive categories. The oncogenetic tree analysis resulted in a reproducible tree with three branches: (1) The first branch was initiated by the methylation of “methylation in tumor” (MINT) sites, predisposing to MSI, methylation of *MLH1* and *TP16*, and *B-Raf* mutation; (2) the second branch was initiated by an *APC* mutation and followed by a *p53* mutation; and (3) the third branch was due to a *K-Ras* mutation and was not followed by any other genetic alteration [31].

By making use of the same primer panel applied in this study *APC*, *K-Ras*, *B-Raf* and *CTNNB1* were found to be mutated in 44, 24, 14 and 4% of sporadic CRC samples analyzed, respectively [20]. If one compares the above-mentioned mutation frequencies in sporadic CRC with the mutation frequencies of *APC*, *K-Ras*, *B-Raf* and *CTNNB1* in serrated lesions and traditional adenomas in this study, it is evident that the mutation frequency along the serrated carcinogenesis pathway increases in the case of *APC*, remains basically unaltered in the case of *K-Ras* as well as *CTNNB1* and decreases in the case of *B-Raf*. Taken together, these results support the view that an altered *APC* gene confers the lesions a growth/selection advantage, which is in line with previous studies showing that *APC* mutations are sufficient for the growth of early colorectal adenomas [26] and that truncating *APC* mutations have dominant effects on proliferation, spindle checkpoint control, survival and chromosome stability [32].

A limited number of lesions in this study carried more than one mutated gene, the most frequent mutation “combinations” being *APC*/*K-Ras* (in three adenomas) and *APC*/*B-Raf* (in one serrated lesion and three adenomas). Moreover, in the present study and in accordance with previous reports [14,33] it has been shown that *B-Raf* as well as *K-Ras* may simultaneously be mutated. However, earlier studies, based on the analysis of DNA isolated from formalin-fixed and paraffin-embedded samples, postulated that *B-Raf* and *K-Ras* mutations were mutually exclusive in precancerous colorectal lesions [15,16,34]. This discrepancy could be due to the fact that, at the time the latter studies were performed, no special kits for the isolation of intact DNA from formalin-fixed samples were available and that the formalin treatment of the samples had led to a partial degradation of DNA, so that certain gene mutations could not be detected. The percentage of low dysplastic lesions carrying any mutations was lower than that of high dysplastic lesions. However, because of the small sample size analyzed in this pilot study, this observation should be viewed with caution, and at the moment there is no clear-cut evidence that precancerous lesions accumulate the mutations detected by our marker panel as the degree of dysplasia increases. Nevertheless, one cannot discard the possibility that further alterations like epigenetic changes, MSI or mutations in different—maybe still unknown—signaling pathways, which can support dysplastic progression, may accumulate with time. Additionally, a genetic predisposition for hypermethylation of gene promoters or environmental poisons might influence the progress of the lesions as well as the lesion type arising [13]. For example, a strong relationship of heavy cigarette smoking with the CpG island methylator phenotype (CIMP) and *B-Raf* mutations, which predispose for the serrated pathway, was reported [35]. If one compares the percentage of colorectal carcinomas carrying more than one mutated gene (14%) determined in our group [20], by making use of the same marker panel applied in this pilot study, with that of serrated lesions and adenomas carrying one mutated gene (Table 2), as well as with that of serrated lesions (3 out of 20, 15%) and adenomas (8 out of 41, 20%) carrying more than one mutated gene, it must be concluded that in the course of the serrated lesion-adenoma-carcinoma sequence, mutations do not accumulate. In the present report 18% of the mutated precancerous lesions (5 adenomas and 1 serrated lesion) showed alterations in both the Wnt and the Ras-Raf-MEK-MAPK pathways. This finding most probably indicates that these lesions were developing through the fusion pathway described by Jass and colleagues [14] rather than being at a more advanced stage of the carcinogenic process.

In the present study about half of the adenomas with a *B-Raf* mutation were located in the proximal colon. In addition, it has recently been reported that the majority of CRC with a mutated *K-Ras* or *B-Raf* gene do in fact arise in the proximal colon [20,36,37]. Taken together, these results support the hypothesis that alterations of the Ras-Raf-MEK-MAPK signaling cascade could play an important role in the development of tumors in the proximal colon. In the case of *APC*, a higher mutation frequency was observed in adenomas arising in the distal colon when compared to those in the proximal colon. This finding is in accordance with a study by Suraweera *et al.* [38], in which significantly more *APC* mutations were detected in microsatellite-stable than in microsatellite-instable tumors. Microsatellite-instable tumors are known to arise more frequently in the proximal colon, whereas microsatellite-stable cancers arise more often in the distal colon and exhibit chromosomal instability [39]. However, one should be aware that the number of samples listed in Table 4 is very low. Hence, the data regarding the correlation between the anatomical location of serrated lesions and traditional adenomas, and the frequency of genetic alterations in these lesions, reveal merely a trend. Future studies involving the analysis of a higher number of potentially precancerous lesions are necessary to confirm the above-mentioned findings.

The mutation frequencies of all four genes analyzed by making use of the conceived primer panel in the present report are within the range of, or even higher than, those of previous studies, in which an individual gene marker was used. Considering the fact that the overall survival of patients with carcinomas detected at stage II or later is still quite low, one must demand that screening markers be capable of detecting nearly all existing stage I carcinomas and as many precancerous lesions as possible. The herein presented marker panel has a sensitivity of up to 65% for the detection of all the precancerous lesions that would usually be removed during routine colonoscopy, in accordance with our aim. In our preceding study [20] the sensitivity of the marker panel regarding the detection of stage I carcinomas was 80%. These results are consistent with the adenoma-carcinoma-sequence and the serrated lesions-serrated carcinoma sequence, in which mutations in the epithelial cells of the colon lead to precancerous lesions and finally to colon cancer. The inflamed tissues used as controls did not contain any mutations, but the number of samples analyzed in this pilot study was too small to deduce the specificity. This should be done in a larger cohort of cases. Taken together, our marker panel, considering defects in two cancer-relevant signaling pathways, seems to be well suited for the screening of precancerous as well as early cancerous lesions of the colon, and therefore might also contribute to cancer prevention. The panel will now be used to detect genetic alterations in stool DNA of patients with precancerous lesions and colorectal carcinomas.

## 4. Experimental

### 4.1. Human Tissue Samples

The present pilot study was designed as a prospective study. Forty-one colorectal adenomas, 20 serrated lesions, and 10 inflamed colon tissue samples from patients that had undergone colonoscopy and associated routine histopathologic assessments were obtained, freshly frozen from the Department of Medicine I at the Charité-Universitätsmedizin Berlin (Berlin, Germany) and the Division of Gastroenterology at the Maria Heimsuchung Caritas-Klinik Pankow (Berlin, Germany), between November 2004 and October 2006. Patients with known hereditary disorders like familial adenomatous polyposis or hereditary nonpolyposis colorectal cancer were excluded. Concerning the inflamed tissues that served as the control group, we excluded all samples with accessory aberrations possibly connected with carcinogenesis like hyperplastic mucosa, any signs of dysplasia, deprivation of goblet cells, as well as tissues from patients with personal history of colorectal adenoma or carcinoma. The routinely removed serrated lesions were not further differentiated and thus may contain sessile serrated adenomas, traditional serrated adenomas and hyperplastic polyps. The mean age of the study population was 66.7 years and did not vary significantly among patients with an adenoma, a serrated lesion or an inflamed colon. Thirty-four tissue samples were from male and 37 from female patients. The study was carried out in compliance with the Helsinki Declaration. Permission for the study was given by the Ethics Commission of the University of Potsdam, Germany (Decision 3/18 taken on July 28, 2004).

### 4.2. Reagents and Kits

Buffer salts were obtained from AppliChem (Darmstadt, Germany) and Carl Roth (Karlsruhe, Germany). PCR reagents were delivered by Bioline (Luckenwalde, Germany), restriction enzymes by Roche Diagnostics (Mannheim, Germany) and New England Biolabs (Frankfurt am Main, Germany). Kits for purification of PCR products (QIAquick^®^ PCR purification kit) and gel extraction (QIAquick^®^ gel extraction kit) were purchased from Qiagen (Hilden, Germany).

### 4.3. DNA Extraction

Genomic DNA was extracted from freshly frozen tissues using a commercial kit (QIAamp DNA Mini Kit, Qiagen). Since *K-Ras* mutation analyses required a high amount of DNA, an aliquot was subjected to whole genome amplification by using Multiple Displacement Amplification (MDA) technology (REPLI-g^®^ Mini Kit, Qiagen) according to the manufacturer's instructions.

### 4.4. Mutation Analysis

Control fragments for use in PCR-SSCP or RFLP analyses were cloned from human carcinoma cell lines with known mutations or were constructed by site-directed mutagenesis. The following mutations were used as positive controls: codon 600 (GTG → GAG) in the *B-Raf* gene; codon 33 (TCT → TAT), codon 41 (ACC → GCC and ACC → ATC), codon 45 (TCT → CCT and TCT → TTT) and *del* codon 45 (deletion of the complete codon) in *CTNNB1*; codon 12 (GGT→ GAT) and codon 13 (GGC →GAC) in *K-RAS*.

**Figure 1.**
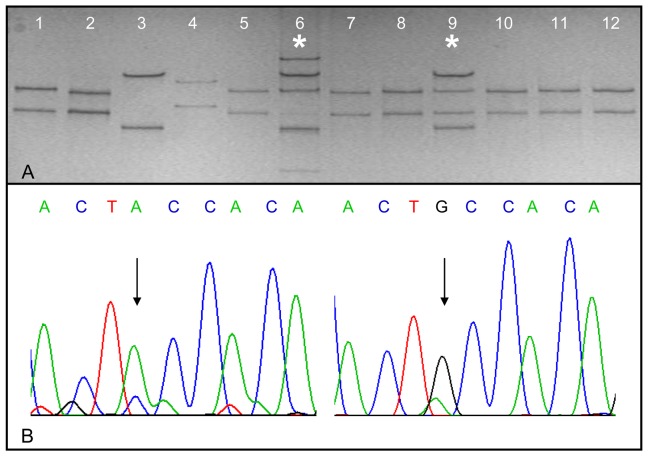
(**A**) PCR-SSCP analysis of the *CTNNB1* gene. Lane 1: wild-type control; Lane 2: mutation control - codon 33 TCT→TAT; Lane 3: mutation control - codon 41 ACC→GCC; Lane 4: mutation control - codon 45 TCT→CCT, Lanes 5–12: human tissue samples No. 69, 48, 37, 41, 68, 16, 44, and 14. Lanes 6 (sample No. 48) and 9 (sample No. 68) exhibit aberrant band motilities (*). (**B**) Sequencing profiles of DNA fragments isolated from SSCP gel. On the left side: wild-type sequence at codon 41. On the right side: shifted band sequence of sample No. 48. Arrows show the transition of wild-type adenine (on the left side) to guanine (on the right side) at the first base position of codon 41.

PCR-SSCP mutation analysis of *B-Raf* and *CTNNB1* was performed as follows: 20 ng of genomic DNA were amplified by conventional PCR with sequence-specific oligonucleotide primers flanking codons 582–638 and 13–71, respectively [19]. The amplicons were purified from a 2% agarose gel. Electrophoresis of 4 μl of the denatured product was performed by using a 16 × 20 cm non-denaturing polyacrylamide gel in 1× TBE (90 mmol/L Tris base, 90 mmol/L boric acid, 2 mmol/L EDTA; pH 8.0) with 20 mmol/L *N*-(2-hydroxyethyl)piperazine-1-*N*′-(2-ethanesulfonic acid). In order to detect the *B-Raf* mutation, a 10% polyacrylamide gel was run at 18 °C with 300 V for 4.5 h. In the case of the *CTNNB1* mutation analysis a 13% gel was run at 26 °C with 250 V for 5 h and an 8% gel at 21 °C with 200 V for 5 h. Gels were silver stained and shifted bands were cut out, reamplified, purified and sequenced. Each sample was analyzed in triplicate. Figure 1 exemplarily shows a SSCP analysis and the corresponding sequencing profile of the shifted band in the case of the *CTNNB1* gene.

**Figure 2.**
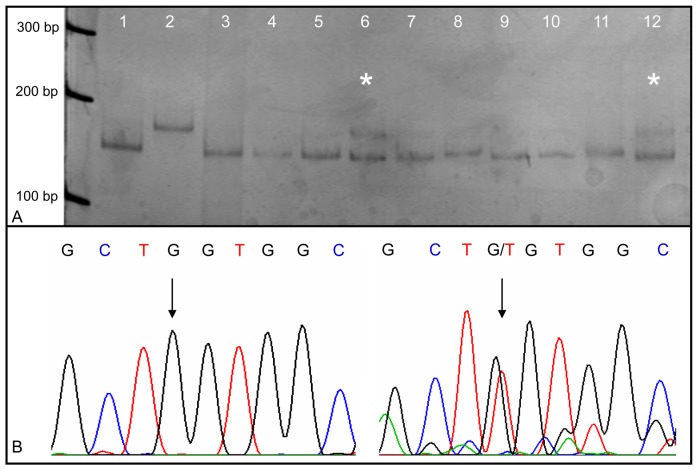
(**A**) PCR-RFLP analysis of the *K-Ras* codon 12 mutation. Lane 1: wild-type control; lane 2: mutation control - codon 12 GGT→GAT; lanes 3–12: human tissue samples No. 44, 64, 6, 18, 13, 43, 1, 4, 69, and 29. Lanes 6 (sample No. 18) and 12 (sample No. 29) exhibit aberrant band motilities (*). (**B**) Sequencing profiles of DNA fragments isolated from polyacrylamide gel. On the left side: wild-type sequence at codon 12. On the right side: shifted band sequence of sample No. 18. Arrows show the transversion of wild-type guanine (on the left side) to thymine (on the right side) at the first base position of codon 12.

*K-Ras* gene mutations were screened by RFLP analysis according to the method described by Schimanski *et al.* [40]. Briefly, 2 μL of the whole genome amplification product (100–300 ng genomic DNA) obtained by using the REPLI-g Mini Kit were PCR-amplified with the oligonucleotide primer pair Ras A and Ras B [19], generating a 166 bp amplicon. The mismatch primer Ras A hybridizing with codons 2–11 of the *K-Ras* gene exon 1 introduces a restriction site for *Bst*XI or *Xcm*I into the amplicon. In case a template is mutated in codon 12 or 13, the restriction site is lost. The subsequent enzymatic restriction of the wild-type *K-Ras* leads to an enrichment of mutated DNA strands. Gel bands, the size of the mutated control fragments, were cut out and sequenced. Figure 2 illustrates a typical RFLP analysis of the *K-Ras* gene and the corresponding sequencing profile of the shifted band.

For mutation analysis of the *APC* gene mutation cluster region (codons 1243–1567), direct DNA sequencing was used. Sequencing was performed by GATC Biotech (Konstanz, Germany).

### 4.5. Statistical Analysis

Fischer's exact test or Pearson's χ^2^ test was applied for comparison of the categorical variables. All tests were two-sided. Probability (*p*) values of less than 0.05 were considered to be statistically significant. All data were analyzed with the statistical software SPSS version 11.0.

## 5. Conclusions

By making use of a recently conceived combination of oligonucleotide primers it could be shown that: (1) In 65% of the analyzed serrated lesions and in 61% of the traditional adenomas of the colon and rectum, at least one of the four genes studied was altered; (2) in 92.3% of the serrated lesions with mutations and in 76% of the adenoma samples with mutations either the Ras-Raf-MEK-MAPK or the Wnt signaling cascade was altered; (3) in 35% of the serrated lesions and in 39% of the adenomas neither the Wnt pathway nor the Ras-Raf-MEK-MAPK pathway were affected. If one takes into account the results of the present study and those of a previous report, in which the primer panel detected 80% of early (UICC I and II) CRC by testing for the same markers, it can be concluded that the conceived primer panel appears suitable for the development of a non-invasive assay for the early detection of CRC.

## Figures and Tables

**Table 1.** Clinicopathologic data of the colorectal lesions analyzed.

	**Inflamed mucosa**		**Serrated lesion**		**Adenoma**	
	**N**	**%**	**N**	**%**	**N**	**%**

Number of samples	10	14.1	20	28.2	41	57.7
Location						
Proximal colon	1	10	8	40	21	51.2
Distal colon	4	40	6	30	13	31.7
Rectum	4	40	4	20	3	7.3
Unknown	1	10	2	10	4	9.8
Degree of dysplasia						
Low grade (mild, moderate)					36	87.8
High grade					3	7.3
Unknown					2	4.9
Histologic typing						
Tubular adenoma					35	85.4
Tubulovillous adenoma					4	9.8
Unknown					2	4.9

**Table 2.** Gene mutation frequency in the colorectal lesions analyzed.

**Tissue type**	***APC***^1^	***K-Ras***^1^	***B-Raf***^1^	***CTNNB1*** 1	**Total** 2

Inflamed mucosa	0/10	0/10	0/10	0/10	0/10
Serrated lesion	2/20 (10)	4/20 (20)	10/20 (50)**	0/20 (0)	13/20(65)***
Adenoma	14/41 (34.2)*	6/41 (14.6)	9/41 (22)	5/41 (12.2)	25/41 (61)***

1 Number of samples with mutations/total number of samples (in parenthesis the percentage of samples carrying the corresponding gene in the mutated form)

2 The total mutation frequency is expressed as the number of samples carrying at least one of the four genes in the mutated form/total number of samples (in parenthesis the percentage of samples carrying at least one of the four genes in the mutated form).

* *APC* mutation frequency is significantly higher in adenomas than in inflamed mucosa (*p* < 0.05, Fischer's exact test).

** *B-Raf* mutation frequency is significantly higher in serrated lesions than in inflamed mucosa (*p* < 0.01, Fischer's exact test).

*** Total gene mutation frequency is significantly higher in serrated lesions as well as in adenomas than in inflamed mucosa (*p* < 0.001, Fischer's exact test).

**Table 3.** Frequency of genetic alterations affecting the Wnt or the Ras-Raf-MEK-MAPK pathway in the colorectal lesions analyzed.

**Tissue type**	**Wnt pathway^1^**	**Ras-Raf-MEK-MAPK pathway^2^**

Inflamed mucosa	0/10	0/10
Serrated lesion	2/20 (10)	12/20 (60)***
Adenoma	17/41 (41.5)	17/41 (34.2)

1 Number of samples with an altered *APC* and/or *CTNNB1* gene/total number of samples (in parenthesis the percentage of samples carrying an altered *APC* and/or *CTNNB1* gene)

2 Number of samples with an altered *K-Ras* and/or *B-Raf* gene/total number of samples (in parenthesis the percentage of samples carrying an altered *K-Ras* and/or *B-Raf* gene)

*** The frequency of genetic alterations affecting the Ras-Raf-MEK-MAPK signaling pathway is significantly higher than that affecting the Wnt pathway (*p* < 0.001, Pearson's χ^2^ test).

**Table 4.** Anatomical location of the colorectal lesions with an altered Wnt or Ras-Raf- MEK-MAPK pathway.

**Location of^1^**	**Wnt pathway**	**Ras-Raf-MEK-MAPK pathway**

Serrated lesions		
proximal colon	0/8	6/8**
distal colon	2/6	3/6
rectum	0/4	2/4

Adenomas		
proximal colon	6/21	9/21
distal colon	8/13*	2/13
rectum	2/3	1/3

1 The location of two serrated lesions and four adenomas was unknown.

* The frequency of genetic alterations affecting the Wnt signaling pathway is significantly higher than that affecting the Ras-Raf-MEK-MAPK signaling pathway in adenomas arising in the distal colon (*p* < 0.05, Fischer's exact test).

** The frequency of genetic alterations affecting the Ras-Raf-MEK-MAPK signaling pathway is significantly higher than that affecting the Wnt signaling pathway in serrated lesions arising in the proximal colon (*p* < 0.01, Pearson's χ^2^ test).

## Supplementary Files

Supplementary File 1 PDF-Document (PDF, 127 KB)
