# Supplementary material for: Detection of up to 65% of Precancerous Lesions of the Human Colon and Rectum by Mutation Analysis of APC, K-Ras, B-Raf and CTNNB1
Source: Cancers (Basel). 2010 Dec 29;3(1):91–105. doi: 10.3390/cancers3010091 (PMC3756351; doi:10.3390/cancers3010091)
Supplement: Supplementary File 1 — PDF-Document (PDF, 127 KB) [file cancers-03-00091-s001.pdf]

Table S1. Mutation status of *APC*, *K-Ras*, *B-Raf* and *CTNNB1* in the colorectal tissues analyzed.

**Sample No.**	**Histological finding**	**Location of lesion**[1]	**Degree of dysplasia**[3]	**Histological typing of ADs**[4]	**Mutations**			
					APC	***K-Ras***	***B-Raf***	***CTNNB1***
1	Inflamed mucosa	9	0					
2	Inflamed mucosa	7	0					
3	Inflamed mucosa	n.a.[2]	0					
4	Inflamed mucosa	6	0					
5	Inflamed mucosa	9	0					
6	Inflamed mucosa	9	0					
7	Inflamed mucosa	1	0					
8	Inflamed mucosa	9	0					
9	Inflamed mucosa	7	0					
10	Inflamed mucosa	7	0					
11	Serrated lesion	7	0					
12	Serrated lesion	9	0					
13	Serrated lesion	1	0				Cd 600 GTG→GAG	
14	Serrated lesion	7	0				Cd 600 GTG→GAG	
15	Serrated lesion	n.a.	0					
16	Serrated lesion	7	0		Cd 1450 CGA→TGA (stop codon)			
17	Serrated lesion	n.a.	0				Cd 600 GTG→GAG	
18	Serrated lesion	4	0			Cd 12 GGT→TGT		
19	Serrated lesion	7	0					
20	Serrated lesion	1	0					
21	Serrated lesion	1	0			Cd 13 GGC→GAC		
22	Serrated lesion	4	0				Cd 600 GTG→GAG	
23	Serrated lesion	2	0					
24	Serrated lesion	7	0		Cd 1465 Del[5] AG		Cd 600 GTG→GAG	
25	Serrated lesion	9	0					
26	Serrated lesion	9	0				Cd 600 GTG→GAG	

27	Serrated lesion	2	0				Cd 600 GTG→GAG	
28	Serrated lesion	4	0				Cd 600 GTG→GAG	
29	Serrated lesion	6	0			Cd 12 GGT→GAT	Cd 600 GTG→GAG	
30	Serrated lesion	9	0			Cd 12 GGT→GAT	Cd 600 GTG→GAG	
31	Adenoma	7	1	1	Cd 1383 Del AT			
32	Adenoma	4	1	1				
33	Adenoma	7	1	1				
34	Adenoma	2/3	1	1				
35	Adenoma	1	1	1				Cd 45 TCT→TTT
36	Adenoma	6	1	1				
37	Adenoma	7	1	1				
38	Adenoma	4	1	1				
39	Adenoma	2	1	1				
40	Adenoma	9	1	1	Cd 1353 GAA→TAA (stop codon)			
41	Adenoma	7	1	1	Cd 1309 GAA→TAA (stop codon)			
42	Adenoma	9	1	1	Cd 1346 Ins A	Cd 12 GGT→AGT		
43	Adenoma	n.a.	1	1				
44	Adenoma	2	2	2			Cd 600 GTG→GAG	
45	Adenoma	1	2	1	Cd 1317 GAA→CAA (glutamic acid → glutamine)			
46	Adenoma	1	1	2	Cd 1465 Del AG		Cd 600 GTG→GAG	
47	Adenoma	1	1	1		Cd 12 GGT→GAT<br>Cd 13 GGC→GAC		
48	Adenoma	2	1	1				Cd 41 ACC→GCC
49	Adenoma	4	1	1			Cd 600 GTG→GAG	
50	Adenoma	6	1	1	Cd 1309-1311 Del 5 bp			
51	Adenoma	1	1	1				
52	Adenoma	n.a.	2	1			Cd 600 GTG→GAG	
53	Adenoma	3	1	1				

54	Adenoma	6	1	1	Cd 1465 Del AG		Cd 600 GTG→GAG	
55	Adenoma	4	1	1				
56	Adenoma	2	1	1	Cd 1556 Ins A	Cd 13 GGC→GAC		
57	Adenoma	4	1	1				
58	Adenoma	6	n.a.	n.a.				
59	Adenoma	6	n.a.	n.a.				Cd 45 TCT→TTT
60	Adenoma	2	1	2			Cd 600 GTG→GAG	
61	Adenoma	n.a.	1	1			Cd 600 GTG→GAG	
62	Adenoma	n.a.	1	1	Cd 1450 Del 7 bp			
63	Adenoma	7	1	1				
64	Adenoma	9	1	1				
65	Adenoma	2	1	1		Cd 13 GGC→GAC	Cd 600 GTG→GAG	
66	Adenoma	7	1	1	Cd 1506 Ins A			Cd 41 ACC→GCC
67	Adenoma	7	1	1	Cd 1410 Ins T			
68	Adenoma	7	1	1	Cd 1556 Ins A	Cd 13 GGC→GAC		Cd 41 ACC→GCC
69	Adenoma	4	1	1		Cd 13 GGC→GAC		
70	Adenoma	2	1	1	Cd 1450 CGA→TGA (stop codon)		Cd 600 GTG→GAG	
71	Adenoma	4	1	1				

[1] Location of lesion: 1: caecum; 2: ascending colon; 3: hepatic flexure; 4: colon transversum; 5: splenic flexure; 6: descending colon; 7: sigmoid; 8: rectosigmoid junction; 9: rectum

[2] n.a., information not available

[3] Degree of dysplasia: 0: no dysplasia; 1: low grade (mild, moderate); 2: high grade

[4] Histologic typing of adenomas: 1: tubular; 2: tubulovillous

[5] Del, deleted; Ins, inserted
